# Supplementary material for: Loss of sphingomyelin synthase-1 does not cause egg retention or locomotion defects in Caenorhabditis elegans
Source: Biol Open. 2026 Mar 23;15(3):bio062520. doi: 10.1242/bio.062520 (PMC13054943; doi:10.1242/bio.062520)
Supplement: Supplementary information [file biolopen-15-062520-s1.pdf]

Dataset 1. *sms-1* allele information

The cDNA and predicted protein sequences in wild-type and the three *sms-1* deletion alleles (*ok2399*, *rp398* and *rp399*) used in this study. Red text = incorrect amino acids incorporated prior to the stop codon (*) in *ok2399*.

> wild-type sms-1 isoform a cDNA

```
ATGAAAATGTCTTGGAATCATCAATACACAAACTATGGATCTATTGCAGACGACAACGGTGA
CGAGGAGAAGGCGGAAAATTCAGAAGGTGCTGCTGCTGAAAAAGTAGAAAAGCAGCACGACG
ACGACGGTGTTGTTGTCCACGAAGAGACTGACGGCGTGGCAAGCAGCCGAAGCAGTCATCAC
GACAAACAAAAACCGGGGGAAACGAAGAAAAGTGGTGACGGAAAAATGGATGATGACGATAT
TATCACAACGGCGAGATCTTCGTCTCGTCGAATCTGTGGTTCGGCGGCCTCGAGTTCTGACT
CTGAAACAGCCGACGACGCCCCGTTGCTACCTGACGAAGGACCATCGCATGCCGTACGACTC
GAAATGCCTGGTGATAAACCAGCAAGTCCCCATGATCGGTTTCCGAAGACACCTCTGAAAAC
GTTGGTTGCGTTTCTGATGCTCGTCGTAGCGGCAGCTGGAAATACAATCACATTATCATGGA
TTCATGAACGATATCCATTGACACCACCACTTCCTGATATTGTGTTCGAGTTAATACCAAAG
ATTCCATGGGGACTTCGATTATGTGAAAATCTTATGATAGGATCATTTGTATCACTTCTTGT
ACTTATTCTCTTTCATAGGCATCGATGGATTGTGCTCCGTCGTCTCTGTTTCATTGGATCAA
TTCTCTATGGTATGAGATGTATCACAATGATGGTTACTCCAGTGCCAAAAGCCGACGAAGAT
TTCGAATGCTCGCCACGTTTCGGTGAAAATGCCACGTTCTCGTTGATTGTGATGCGAGGTGT
TTGGAGCATGTTCGGGCTCGGTCTCAACTTATTCGACAATCAAAAAGTCGTTTTGTGTGGAG
ACTACATTTACAGTGGACACACACTTGTGCTCGTCGTATCGGCTCTTTTCATTGGAGAATAC
TCTCCTCGACGATTCTACATTCTTCACTGGCTGTCATGGTTAGTGTGTTCCGTTGGTGTGAT
CTTTCTAGTTCTATCACACGGACACTACACTATTGATGTGATACTTTCATATTTCGCTTGTA
CACGAGTATTCTGGGCATATCATACACAGGCTGCACATCCATCCATTCGGCTTTCTGTACAA
AATCATCAAGCCAAAGAGTTTTGGTTCCCATTGTTGCGATGGTTTGAAGGAGACATACGGCG
ACCTGTTCCACGAAGATTCGATTGTCCAATCTCGTATTCACAAGTGTGTAATGCTTTCCGGC
GAGTTCGTCCACGTGGCAGAAATGGAGCTGCACGACCTGCTTTCGAATGA
```

> wild-type sms-1 isoform a 429aa

```
MKMSWNHQYTNYGSIADDNGDEEKAENSEGAAAEKVEKQHDDDGVVVHEETDGVASSRSSHH
DKQKPGETKKSGDGKMDDDDIITTARSSSRRICGSAASSSDSETADDAPLLPDEGPSHAVRL
EMPGDKPASPHDRFPKTPLKTLVAFLMLVVAAAGNTITLSWIHERYPLTPPLPDIVFELIPK
IPWGLRLCENLMIGSFVSLLVLILFHRHRWIVLRRLCFIGSILYGMRCITMMVTPVPKADED
FECSPRFGENATFSLIVMRGVWSMFGLGLNLFDNQKVVLCGDYIYSGHTLVLVVSALFIGEY
SPRRFYILHWLSWLVCSVGVIFLVLSHGHYTIDVILSYFACTRVFWAYHTQAAHPSIRLSVQ
NHQAKEFWFPLLRWFEGDIRRPVPRRFDCPISYSQVCNAFRRVRPRGRNGAARPAFE
```

Biology Open • Supplementary information

> sms-1(rp398)isoform a cDNA

ATGAAAATGTCTTGGAATCATCAATACACAAACTATGGATCTATTGCAG

> sms-1(rp398)isoform a

MKMSWNHQYTNYGSIA*

> sms-1(rp399)isoform a cDNA

ATGAAAATGTCTTGGAATCATCAATACACAAACTATGGATCTATTGCAG

> sms-1(rp399)isoform a

MKMSWNHQYTNYGSIA*

> sms-1(ok2399)isoform a cDNA

ATGAAAATGTCTTGGAATCATCAATACACAAACTATGGATCTATTGCAGACGACAACGGTGA
CGAGGAGAAGGCGGAAAATTCAGAAGGTGCTGCTGCTGAAAAAGTAGAAAAGCAGCACGACG
ACGACGGTGTTGTTGTCCACGAAGAGACTGACGGCGTGGCAAGCAGCCGAAGCAGTCATCAC
GACAAACAAAAACCGGGGGAAACGAAGAAAAGTGGTGACGGAAAAATGGATGATGACGATAT
TATCACAACGGCGAGATCTTCGTCTCGTCGAATCTGTGGTTCGGCGGCCTCGAGTTCTGACT
CTGAAACAGCCGACGACGCCCCGTTGCTACCTGACGAAGGACCATCGCATGCCGTACGACTC
GAAATGCCTGGTGATAAACCAGCAAGTCCCCATGATCGGTTTCCGAAGACACCTCTGAAAAC
GTTGGTTGCGTTTCTGATGCTCGTCGTAGCGGCAGCTGGAAATACAATCACATTATCATGGA
TTCATGAACGATATCCATTGACACCACCACTTCCTGATATTGTGTTCGAGTTAATACCAAAG
ATTCCATGGGGACTTCGATTATGTGAAAATCTTATGATAGGATCATTTGTATCACTTCTTGT
ACTTATTCTCTTTCATAGCGTCGTCTCTGTTTCATTGGATCAATTCTCTATGGTATGAGATG
TATCACAATGATGGTTACTCCAGTGCCAAAAGCCGACGAAGATTTCGAATGCTCGCCACGTT
TCGGTGAAAATGCCACGTTCTCGTTGATTGTGATGCGAGGTGTTTGGAGCATGTTCGGGCTC
GGTCTCAACTTATTCGACAATCAAAAAGTCGTTTTGTGTGGAGACTACATTTACAGTGGACA
CACACTTGTGCTCGTCGTATCGGCTCTTTTCATTGGAGAATACTCTCCTCGACGATTCTACA
TTCTTCACTGGCTGTCATGGTTAGTGTGTTCCGTTGGTGTGATCTTTCTAGTTCTATCACAC
GGACACTACACTATTGATGTGATACTTTCATATTTCGCTTGTACACGAGTATTCTGGGCATA
TCATACACAGGCTGCACATCCATCCATTCGGCTTTCTGTACAAAATCATCAAGCCAAAGAGT
TTTGGTTCCCATTGTTGCGATGGTTTGAAGGAGACATACGGCGACCTGTTCCACGAAGATTC
GATTGTCCAATCTCGTATTCACAAGTGTGTAATGCTTTCCGGCGAGTTCGTCCACGTGGCAG
AAATGGAGCTGCACGACCTGCTTTCGAATGA

> sms-1(ok2399)isoform a

MKMSWNHQYTNYGSIADDNGDEEKAENSEGAAAEKVEKQHDDDGVVVHEETDGVASSRSSHH
DKQKPGETKKSGDGKMDDDDIITTARSSSRRICGSAASSSDSETADDAPLLPDEGPSHAVRL
EMPGDKPASPHDRFPKTPLKTLVAFLMLVVAAAGNTITLSWIHERYPLTPPLPDIVFELIPK
IPWGLRLCENLMIGSFVSLLVLILFHSVVSVSLDQFSMV*

Fig. S1. ***sms-1*** **allele information**

The cDNA and predicted protein sequences in wild-type and the three *sms-1* deletion alleles (*ok2399*, *rp398* and *rp399*) used in this study. Red text = incorrect amino acids incorporated prior to the stop codon (*) in *ok2399*.

Biology Open • Supplementary information

Table S1. Reagent information and source data.

Available for download at

https://journals.biologists.com/bio/article-lookup/doi/10.1242/bio.062520#supplementary-data

Biology Open • Supplementary information
